# Anthropometric Indicators of the Cardiometabolic Risk, Muscle Strength, and Functional Capacity of Schoolchildren with Intellectual Disabilities during Lockdown in Chile

**DOI:** 10.3390/children9091315

**Published:** 2022-08-29

**Authors:** Claudio Farías-Valenzuela, Gerson Ferrari, Sebastián Espoz-Lazo, Paloma Ferrero-Hernández, Emilio Jofré-Saldia, Sebastián Álvarez-Arangua, Carlos Poblete-Aro, Andrés Godoy-Cumillaf, Cristian Cofre-Bolados, Pedro Valdivia-Moral

**Affiliations:** 1 Instituto del Deporte, Universidad de las Américas, Santiago 9170022, Chile; 2 Department of Didactics of Musical, Plastic and Corporal Expression, Faculty of Education, University of Granada, 18071 Granada, Spain; 3 Sciences of Physical Activity, Sports and Health School, University of Santiago of Chile (USACH), Santiago 9170022, Chile; 4 Human Performance Lab, Education, Physical Activity and Health Research Unit (GEEAFyS), Universidad Católica del Maule, Talca 3460000, Chile; 5 Facultad de Ciencias para el Cuidado de la Salud, Universidad San Sebastian, Lota 2465, Providencia 7510157, Chile; 6 Facultad de Educación y Cultura, Universidad SEK, Santiago 7520318, Chile; 7 Excercise and Rehabilitation Sciences Laboratory, School of Physical Therapy, Faculty of Rehabilitation Sciences, Universidad Andres Bello, Santiago 7591538, Chile; 8 Escuela de Enfermería, Universidad Santo Tomás, Santiago 8320000, Chile; 9 Grupo de Investigación en Educación Física, Salud y Calidad de Vida, Universidad Autónoma de Chile, Temuco 478000, Chile

**Keywords:** COVID-19, lockdown, physical condition, functional capacity, muscle strength, handgrip strength, intellectual disability, schoolchildren, cardiometabolic risk

## Abstract

Lockdown due to the COVID-19 pandemic has negatively impacted the social, psychological, and physical well-being of the world population. In the case of people with intellectual disabilities, the impact of lockdown on their physical condition and functionality is not completely clear. This study aimed to determine the effects of COVID-19 lockdown on the anthropometric indicators of cardiometabolic risk, muscle strength, and functionality on schoolchildren with intellectual disabilities. The sample was composed of 132 students of both sexes (*n* = 74 pre-lockdown; *n* = 58 lockdown) belonging to two special education centers from the Metropolitan Region of Santiago, Chile. Our results showed significant reductions (*p* ≤ 0.05) in absolute and relative handgrip strength, as well as in functionality, when comparing pre-lockdown and lockdown measurements, with a greater loss in girls than boys. The design and implementation of physical exercise programs centered on strength training are necessary for the physical and functional reconditioning of this population. These programs need to be implemented in special education centers considering the general well-being, quality of life and work needs of people with intellectual disabilities.

## 1. Introduction

Since the beginning of the COVID-19 pandemic, governments across the world have been forced to use prolonged lockdowns to curb the spread of coronavirus and safeguard the world population [1]. This has led to a deterioration of the general health of people, with repercussions in the social, psychological, and physical dimensions of human beings [2]. In this context, voluntary exercise has become a survival mechanism for the species as a decrease in it is related to functional and organic degradation across age, gender, and intellectual capacity [3]. This problem is even more noticeable in people with intellectual disabilities (ID), who before lockdown already presented high overweight and obesity indexes [4] and low levels of physical activity [5] and muscle strength, factors that are linked to a deterioration of functionality in daily activities [6]. These determinants also condition the performance of instrumental and daily life skills, compromising autonomy and independence [7]. People with ID present lower levels of physical condition than people without disabilities [8], which is related to an increase in the likelihood of early cardiometabolic diseases as a consequence of the physical condition levels and the predominance of some sedentary behavior in this population [9]. Before the COVID-19 pandemic, sex-based differences in the anthropometric measures of cardiometabolic disease, muscle strength, and functionality of adolescents with ID were already reported, affecting women more than men [10]. The early alteration of these components from school age in people with ID could affect different health dimensions, as well as autonomy during the adult stage [11]. Likewise, this impacts the physical and functional support that special education institutions provide for the labor world [12]. Movement restrictions due to lockdowns exacerbated even more the gap in the access to schooling services and the use of institutions specialized in people with ID, negatively impacting the quality of life of this population [13]. Compared to the absence of lockdown, the pandemic reduced by up to 61% the levels of physical activity in people with ID [14]. In this sense, de-schooling due to the COVID-19 pandemic decreased the possibilities of socialization through group activities and interactions among schoolchildren, heightening non-compliance with the recommendations of physical activity for this population, which suggest an average of 60 min/day of physical activity at moderate and vigorous intensity in children under 18 years of age [15]. In addition, a decrease in explosive muscle actions related to strength and power were observed [16], together with muscle disuse due to a lack of motor stimuli [17] and exercise deficit disorder, accompanied by problems such as pediatric dynapenia and physical illiteracy [18]. Studies around the world have highlighted the negative impact on physical fitness during COVID-19 in schoolchildren [19], with an increase in cardiometabolic risk [20] and a reduction in muscular fitness [21] as consequences. However, changes in the dimensions of physical fitness by sex in people with ID are not fully understood. The originality and novelty of this study is supported by the fact that current scientific evidence is scarce in Chile and the world in declaring the effects of confinement on physical fitness and functionality in schooled people with ID. The aim of this study is to determine the effects of COVID-19 lockdowns on anthropometric indicators of cardiometabolic risk, muscle strength, and functional capacity in schoolchildren of both sexes with ID from Santiago de Chile.

## 2. Materials and Methods

### 2.1. Design and Participants

This was a retrospective cross-sectional study. We considered a statistical relevance of 80% with a 5% limit error and 95% confidence interval, required for a sample size of 44 subjects [22]. The sample was composed of schoolchildren (*n* = 81 boys and *n* = 51 girls), who formed two independent mixed groups, one pre-lockdown group (*n* = 74) and one lockdown group (*n* = 58). Participants were selected by convenience. All of them attended two special education schools from Santiago, in the Metropolitan Region of Chile. Parents and/or guardians of participants signed an informed consent authorizing the voluntary participation of students in this study. The study followed the Declaration of Helsinki guidelines for research on human beings [23], and was approved by the ethics committee of Universidad de Granada, under the code 2052/CEIH/2021.

The inclusion criteria considered were the following: light to moderate intellectual disability diagnosis based on the Wechsler Intelligence Scale for Children or WISC III [24], and Wechsler Adult Intelligence Scale or WAIS IV [25]; attending a special education center; active participation in the physical education classes either face-to-face or remotely (minimum of 90 min, once per week), independent mobility and a medical certificate of health in order to participate. The following exclusion criteria were used: severe-deep intellectual disability, dependence in performing motor tasks, negative results in anthropometric tests, incomplete muscle and functional strength tests, wheelchair dependence, and being under or equal to 12 years of age.

In total, 115 students were assessed pre-lockdown and 88 during lockdown. Forty-one students and 30 students were excluded from the pre-lockdown and lockdown groups for not meeting the inclusion criteria. Data were collected from participants during the first stage, between August and November 2019 in the context of the project “Inclusión en Movimiento” (Inclusion in Motion). The second stage was conducted 2 years later, between August and November 2021, during the first post-lockdown stage in Chile, under the project “Ludoinclusión 19”. Both projects belonged to the Vice-rector’s Office for Community Outreach (VIME, in Spanish) of Universidad de Santiago de Chile.

### 2.2. Variables and Instruments 

#### 2.2.1. Anthropometry and Cardiometabolic Risk

The body weight of schoolchildren was recorded in kilograms (kg) and their height in centimeters (cm). Both measurements were conducted using a digital scale with a SECA brand stadiometer, model 206. Waist circumference (WC) was measured in cm with a CESCORF brand inextensible metal tape measure, calibrated in centimeters of millimetric graduation. To conduct the assessment, the Moreno-González protocol [26] was employed, which considered the middle point of the distance between the costal inferior margin and the superior margin of the iliac crest. Of the anthropometric measures, the body mass index (BMI: body weight ((kg)/(m^2^)) and the waist to height ratio (waist circumference (cm)/height (cm)) were calculated. WC, BMI, and WHtR were considered anthropometric markers of cardiometabolic risk in schoolchildren with ID [27].

#### 2.2.2. Muscle Strength

To assess isometric strength, handgrip strength (HGS) was measured with a hydraulic dynamometer from the brand Baseline^®^ LiTE^®^ using the American College Sports Medicine guidelines [28]. The protocol employed in schoolchildren consisted of two attempts with each upper limb after familiarization with the instrument. Attempts were separated by one minute of pause. Finally, the average between both attempts was considered the final value of HGS for each limb [29]. Subsequently and once absolute HGS was recorded, relative HGS was calculated (HGS (kg)/body weight (kg)). Dynamic strength was measured with the contact platform Cronojump^®^. Students performed a countermovement jump (CMJ) from the Bosco test. Participants were instructed to jump as high as possible on the contact platform, without separating their hands from their hips, imitating the movement performed by the evaluator [27]. Each participant was allowed two trial attempts. Then, the height of the two jumps was recorded considering the average between the two jumps the final measurement of CMJ [10]. 

#### 2.2.3. Functional Capacity

To assess functional capacity, three tests were conducted: 

(a) Timed Up and Go test (TUG): the test consisted of standing up from a chair and walking as fast as possible going around the obstacle located at three meters, turning around and going back to the initial position. Previously, the individual had to remain seated on a chair without resting his arms, keeping his back in contact with the backrest and the feet touching the ground until the evaluator’s signal to stand up. Before the assessment, participants had two attempts to familiarize themselves with the instrument. During the test, participants were encouraged to perform the action at the maximum speed possible. The evaluator recorded the total time required to complete the course [30].

(b) Five Times Sit to Stand Test (5STS): which consisted of performing five continuous sets of sitting down and standing up in the shortest time possible. A chair of 43 (cm) in height was employed. Seated participants were asked to move forward in the chair until their feet were on the ground, as well as crossing their upper limbs over their chest. Subsequently, participants were asked to stand up adopting that position and repeating the action five times as fast as they could. Two trial attempts were performed. The chronometer was started at the moment of the verbal cue “start!” and stopped at the end of the fifth position when the participant was sitting. The time employed was recorded as the score of the participant [31].

(c) 4 × 10 m speed and agility: The test consisted of running a distance of 40 (m) divided in four segments of 10 m in the shortest time possible. Two parallel lines of 10 m apart were demarcated. An evaluator (A) was positioned at the exit line and a second evaluator (B) at the opposite line, who was in charge of guiding and encouraging each participant to complete the lap and finish the test. Each time participants reached the ends where the evaluators were positioned (every 10 m), they touched the hand of the evaluator. Participants familiarized themselves with the lap before the final assessment. Evaluator (A) clocked the time that took participants to complete the test in seconds and hundredths of a second [29]. Then, the 40 m run was divided by the total test time in seconds to determine the average race speed (m/s) (ARS: distance (m)/time (s)).

### 2.3. Statistical Analysis

To compare the normality of the studied variables, the Kolmogorov–Smirnov test was used. The descriptive statistics were represented using mean and standard deviation, as well as median and interquartile intervals (IQR: 25th and 75th). To establish comparisons between the independent samples of the variables with parametric distribution (CMJ and ARS), a T-Student test was used for the independent variables, while the comparisons of the variables with non-parametric distributions (Weight, BMI, WC, WHtR, AR-HGS, AL-HGS RR-HGS, RL-HGS, TUG, 5STS, Agility 4 × 10 m) were conducted with the U Mann–Whitney test. The statistical package employed was the SPSS V27 software (SPSS Inc., IBM Corp., Armonk, New York, NY, USA). A significance level of 5% was adopted.

## 3. Results

Descriptive statistics are shown in Table 1 in the form of means and standard deviation, median, and interquartile interval (25th and 75th). The schoolchildren sample was described in the previous table (*n* = 132). Schoolchildren participating in the study (*n* = 74) in the pre-lockdown period were 44 boys (59.4%) and 30 girls (40.6%). During lockdown, participants (*n* = 58) were 37 boys (63.7%) and 21 girls (36.3%). 

**Figure 1:**
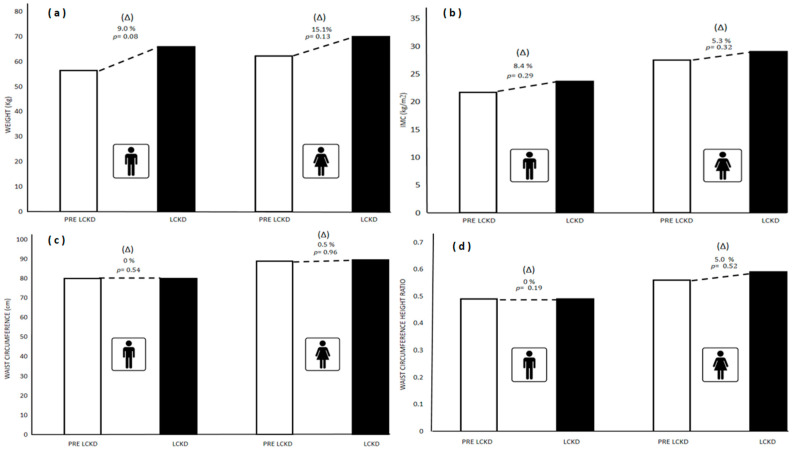
Differences in body weight, BMI, waist circumference, and waist-to-height ratio pre-lockdown and during lockdown in schoolchildren with ID in both sexes. (**a**) Body weight; (**b**) BMI; (**c**) waist circumference; (**d**) waist-to-height ratio. Data are presented as medians, significance value *p* ≤ 0.05 used for the Mann–Whitney U test. PRE LCKD: Pre Lockdown; LCKD: During Lockdown.

Figure 1 presents the differences between medians and (Δ) % in anthropometric and cardiometabolic risk measures pre-lockdown and during lockdown for schoolchildren with ID in both sexes. No significant differences were found in body weight (kg) between boys (Pre-lockdown = 56.40; Lockdown = 66.00; ↑9%; *p* = 0.08) and girls (Pre-lockdown = 62.25; Lockdown = 70.00; ↑15.1%; *p* = 0.13); BMI (kg/m^2^) in boys (Pre-lockdown = 21.67; Lockdown = 23.67; ↑8.4%; *p* = 0.29) and girls (Pre-lockdown = 27.48; Lockdown = 29.03; ↑5.3%; *p* = 0.32); WC (cm) in boys (Pre-lockdown = 80.00; Lockdown = 80.00; 0%; *p* = 0.54) and girls (Pre-lockdown = 89.00; Lockdown = 89.50; ↑0.5%; *p* = 0.96); Waist-to-height ratio in boys (Pre-lockdown = 0.49; Lockdown = 0.49; 0%; *p* = 0.19) and girls (Pre-lockdown = 0.56; Lockdown = 0.59; ↑5.0%; *p* = 0.52)

**Figure 2:**
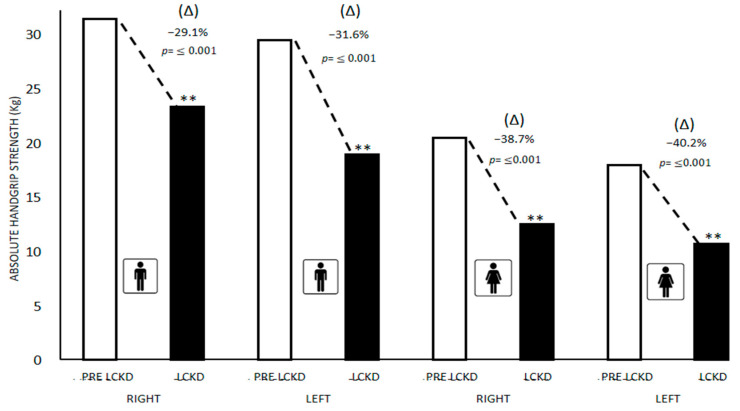
Differences in absolute right and left arm handgrip strength pre-lockdown and during lockdown in schoolchildren with ID in both sexes. Data are presented as medians. ** Significance value *p* = ≤ 0.05 for Mann–Whitney U test. PRE LCKD: Pre Lockdown; LCKD: During Lockdown.

Figure 2 presents the differences between the medians and (Δ) % in the absolute handgrip strength of the right and left arms pre-lockdown and during lockdown in schoolchildren with ID in both sexes. Significant differences (*p* ≤ 0.05) were found in boys between the absolute handgrip strength (kg) of the right arm (Pre-lockdown = 31.50; Lockdown = 22.55; ↓29.1%; *p* ≤ 0.001) and left arm (Pre-lockdown = 29.50; Lockdown = 19.00; ↓31.6%; *p* ≤ 0.001). In girls, significant differences were found between the absolute handgrip strength (kg) of the right arm (Pre-lockdown = 20.50; Lockdown = 12.55; ↓38.7%; *p* ≤ 0.001) and left arm (Pre-lockdown = 18.00; Lockdown = 10.75; ↓40.2%; *p* ≤ 0.001). 

**Figure 3:**
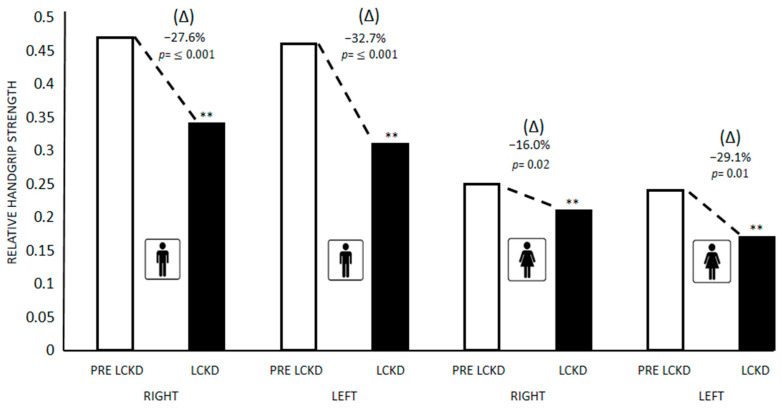
Differences in absolute right and left arm handgrip strength pre-lockdown and during lockdown in schoolchildren with ID in both sexes. Data are presented as medians. ** Significance value *p* ≤ 0.05 for Mann–Whitney U test. PRE LCKD: Pre Lockdown; LCKD: During Lockdown.

Figure 3 presents the differences between the medians and (Δ) % in the relative handgrip strength of the right and left arms pre-lockdown and during lockdown in schoolchildren with ID in both sexes. Significant differences (*p* ≤ 0.05) were found in boys between the right relative handgrip strength of the right arm (Pre-lockdown = 0.47; Lockdown = 0.34; ↓27.6%; *p* ≤ 0.001) and left arm (Pre-lockdown = 0.46; Lockdown = 0.31; ↓37.7%; *p* ≤ 0.001). In girls, significant differences were found between the absolute handgrip strength (kg) of the right arm (Pre-lockdown = 0.25; Lockdown = 0.21; ↓16.0%; *p* = 0.02) and left arm (Pre-lockdown = 0.24; Lockdown = 0.17; ↓29.1%; *p* = 0.01). 

**Figure 4:**
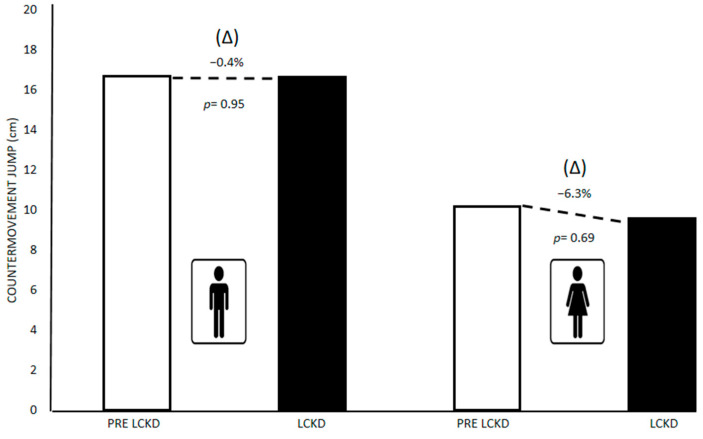
Differences in countermovement jump (CMJ) pre-lockdown and during lockdown in schoolchildren with ID in both sexes. Data are presented as means. Significance value *p* ≤ 0.05 for T-Student test for independent samples. PRE LCKD: Pre Lockdown; LCKD: During Lockdown.

Figure 4 presents the differences between the mean and (Δ) % of CMJ (cm) pre-lockdown and during lockdown in schoolchildren with ID in both sexes. No significant differences were found in CMJ in boys (Pre-lockdown = 16.71; Lockdown= 16.74; ↓0.4%; *p* = 0.95), and girls (Pre-lockdown = 10.20; Lockdown = 9.59; ↓6.3%; *p* = 0.69)

**Figure 5:**
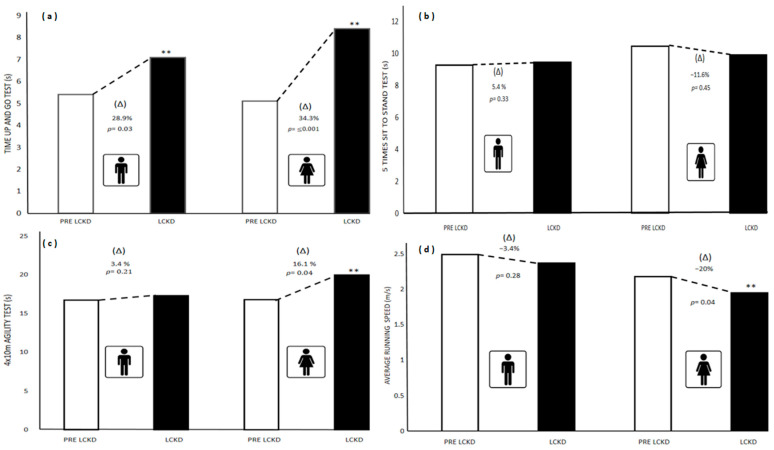
Differences in Time Up and Go test, five time sit to stand test, 4 × 10 m agility test, average running speed pre-lockdown and during lockdown in schoolchildren with ID in both sexes. (aTime up and go test; (**b**) 5 times sit to stand test; (**c**) 4 × 10 m test; (**d**) average running speed. (**a**–**c**) is presented as median and (**d**) and mean. ** Significance value *p* = ≤0.05 for Mann–Whitney U test and T-Student test for independent samples, respectively. PRE LCKD: Pre Lockdown; LCKD: During Lockdown.

Figure 5 presents the differences between the medians and (Δ) % of the functional tests Time Up and Go, STS5 and 4 × 10 m test, and the mean and (Δ) % of the average running speed pre-lockdown and during lockdown in schoolchildren with ID in both sexes. Significant differences (*p* ≤ 0.05) were found in the TUG test (s) for both sexes. Boys (Pre-lockdown = 5.40; Lockdown = 7.49; ↑28.9%; *p* = 0.03); girls (Pre-lockdown = 5.51; Lockdown = 8.39; ↑34.3%; *p* ≤ 0.001). In the agility 4 × 10 m test (s), significant differences were observed only in girls. Boys (Pre-lockdown = 16.71; Lockdown = 17.31; ↑3.4%; *p* = 0.21); girls (Pre-lockdown = 16.76; Lockdown = 19.99; ↑16.1%; *p* = 0.04). Significant differences in ARS (m/s), were also recorded in girls. Boys (Pre-lockdown = 2.49; Lockdown = 2.37; ↓3.4%; *p* = 0.28); girls (Pre-lockdown = 2.18; Lockdown = 1.95; ↓20%; *p* = 0.04). No differences were observed in the STS5 test (s) for both sexes. Boys (Pre-lockdown = 8.48; Lockdown = 8.97; ↑5.4%; *p* = 0.33) and girls (Pre-lockdown = 10.86; Lockdown = 9.59; ↓ 11.6%; *p* = 0.45).

## 4. Discussion

The results of the present study show a reduction in the muscle strength of absolute and relative handgrip in both sexes, more so in girls than boys in the case of functionality tests. No significant changes were observed in the anthropometric indicators of cardiometabolic risk in both sexes.

Our findings demonstrate an increase in body weight when comparing average weight at pre-lockdown and during lockdown (6.1 kg in boys and 11.43 kg in girls, respectively). However, these results were not significant possibly due to data dispersion. Contrary to the results of the study by Mosbah et al. [32], who indicated that lockdown reduced body weight in a sample of adults with Prader Willi syndrome in France, these findings may have been influenced by the lifestyle of families and their approach to the care of people with ID, which varies significantly in Europe and South America, therefore affecting the quality of life of this population [33]. One of the anthropometric measures that was found to be unaffected was WC, with no significant changes when comparing pre-lockdown and lockdown values in both sexes. The above is in agreement with the study by Ramos-Alvarez et al. [34] in a sample of Spanish children assessed during the pandemic in Spain. BMI results in this study showed increases in the indicator but without significant differences. Likewise, a study with university students established that almost 50% of the sample did not suffer BMI variations due to the COVID-19 pandemic [35]. Our results also agree with the results published by Chang et al. [36], who found an increase in body weight and BMI in children and adolescents, without significant increments in these age groups with comorbidities. In the same line, a study with similar characteristics in a sample of 40 children with overweight and obesity did not show significant BMI increases pre- and post-lockdown either [37]. Obesity and overweight were highly prevalent in people with intellectual disabilities since before the lockdown [38]. These conditions could be factors that determined the results obtained. Although no significant changes were observed in the anthropometric measures of cardiometabolic risk, these indicators increased more in girls than boys. These results are contrary to those obtained by Maltoni et al. [39], who detected an increase in body weight of 3.8 kg in men and 1.2 kg in women. Body weight increases affected other anthropometric measure such as BMI and the waist-to-height ratio. The increase in these cardiometabolic risk indicators could be related to a sedentary lifestyle and the lack of physical activity during the pandemic. Regarding the above mentioned, the influence of physical activity levels during lockdown on people with ID may have been a decisive factor to explain the differences in body weight gains of boys and girls, which were not quantified in this study. 

As for muscle strength, a significant reduction in the levels of absolute handgrip strength was observed in Chilean schoolchildren with ID in both sexes. These findings align with the inverse association between time spent in sedentary behavior and functionality, as sedentary habits can lead to neuromuscular function, de-enervation of muscle fibers, and loss of muscle strength, power and mass, which is detrimental for the functional capacity of this population [40]. In addition, the results of this study indicate that in both boys and girls with ID, lockdown increased the body mass of individuals, which in addition to the decrease in absolute handgrip strength, affects the relative levels of handgrip strength. Increments in body weight and reduction in handgrip strength from childhood stages could affect the health of people with ID during adulthood. A study with about 10,000 adults without ID showed an inverse association between high levels of relative handgrip strength and the reduction in the risk of diabetes, hypertension, hyperglycemia, hypertriglyceridemia, low HDL-cholesterol levels, and physical disability [41]. In this sense, fluctuations in relative handgrip strength in schoolchildren can be used as predicting measure for sarcopenic obesity in children [42]. Therefore, it is imperative to control the nutritional state and avoid muscle disuse from school stages in people with ID. Interventions based on the increase in weekly minutes of physical activity from school age could be an alternative for improving muscle strength [43], as well as structuring training programs through recreational activities to improve the body composition and control of weight in schoolchildren with ID [44]. It should be noticed that the population under study constantly face a variety of physical, motor, and functional challenges along their lives, and therefore long periods of lockdown or sedentary activities can be highly detrimental to their neuromuscular and cardiometabolic health. In this sense, handgrip strength is a sensitive-change parameter. Farias-Valenzuela et al. [45] determined that lower levels of relative handgrip strength were related to an increase in anthropometric markers of cardiometabolic risk such as BMI, WC, and WHtR in a sample of 138 schoolchildren of both sexes, with this association stronger in girls than boys. Studies on people with ID have linked high levels of absolute handgrip strength with functional capacity and performance in tests consisting of sitting down and standing up from a chair, agility, jumping and running [6]. Few studies have referred to the effect of lockdown on functional capacity. Andreu-Caravaca et al. [46] studied a sample of 18 people with multiple sclerosis, for whom lockdown did not affect their capacity of standing and sitting from a chair, measured through the 5tsr test; however, lockdown did compromise the time they spent performing the TUG test, increasing execution time as compared to pre-lockdown. These results are in agreement with our study. An increase in the execution time of this test could be related to a risk in falls [47] and a rise in the probability of functional dependence in people with ID [48].

Among the limitations of the study is the design. First, this was not a study with related samples, which was justified by the health context of lockdown and the difficulties to access schools and people with ID. Another limitation is that the sample did not consider a differentiation by syndromes associated with intellectual disability given the low frequency of cases in each category, as well as the influence of family lifestyle and physical activity levels in lockdown. However, few studies in the world and Chile have presented information about the effects of lockdown on people with ID, so this study is pioneering in that it quantified the changes in anthropometric indicators of cardiometabolic risk, muscle strength, and functionality of schoolchildren with ID. In this sense, this study contributes relevant data and orients the guidelines that government programs should adopt in the case of people with ID. In addition, it guides physical educators and special schools in terms of assessments to apply and the design of objectives for the short, medium and long term in order to focus their intervention on strength training and functional capacities of people with ID.

## 5. Conclusions

Two years of lockdown due to the COVID-19 pandemic reduced the absolute handgrip strength, relative handgrip strength, and functional capacity of Chilean schoolchildren with intellectual disabilities, affecting girls more than boys. The promotion of strategies and the design of specific interventions from school age are essential for mitigating the loss of strength of people with ID. In this way, strength training represents a strategy for alleviating the negative effects derived from prolonged periods of physical inactivity as well its repercussions in the functionality of people with ID. 

## Figures and Tables

**Table 1:** Anthropometric, physical, and functional characteristics of Chilean schoolchildren with intellectual disabilities of both sexes, before and after the COVID-19 lockdown.

Variables	Pre-Lockdown			Lockdown		
	Total (*n* = 74)	Boys (*n* = 44)	Girls (*n* = 30)	Total (*n* = 58)	Boys (*n* = 37)	Girls (*n* = 21)
**Age (years)**						
Mean (SD)	16.90 (3.44)	16.02 (3.31)	16.86 (3.20)	17.60 (3.56)	17.24(3.60)	17.21 (3.48)
Median (IQR)	15.00 (14.00–19.00)	15.00 (14.00–17.75)	15.50 (14.75–20.00)	17.00 (14.75–20.25)	17.00 (14.00–20.00)	17.00 (15.00–21.00)
**Weight (kg)**						
Mean (SD)	65.85 (19.64)	61.22 (18.41)	64.07 (17.12)	70.28 (21.06)	67.32 (18.24)	75.50 (24.90)
Median (IQR)	59.45 (48.98–71.00)	56.40 (48.22–69.75)	62.25 (50.50–73.75)	67.50 (52.75–84.95)	66.00 (51.50–81.50)	70.00 (56.50–96.50)
**Height (m)**						
Mean (SD)	1.59 (0.12)	1.61 (0.12)	1.50 (0.09)	1.62 (0.11)	1.66 (0.10)	1.56 (0.11)
Median (IQR)	1.58 (1.46–1.64)	1.63 (1.53–1.71)	1.48 (1.44–1.58)	1.65 (1.55–1.71)	1.69 (1.57–1.73)	1.59 (1.46–1.64)
**BMI (kg/m**^**2**^)						
Mean (SD)	26.14 (7.82)	23.50 (6.54)	28.59 (7.25)	26.88 (8.52)	24.36 (6.01)	31.33 (10.44)
Median (IQR)	23.77 (20.17–29.37)	21.67 (18.14–26.45)	27.48 (23.19–31.53)	25.68 (21.11–32.27)	23.67 (19.71–27.73)	29.03 (24.09–38.17)
**WC (cm)**						
Mean (SD)	84.51 (18.02)	80.66 (14.98)	91.30 (21.11)	86.17 (17.05)	84.44 (15.00)	91.90 (17.25)
Median (IQR)	83.9 (71.37–93.42)	80.00 (70.50–91.75)	89.00 (79.75–103.50)	83.50 (76.38–98.25)	80.00 (74.00–93.75)	89.50 (76.87–103.00)
**WHtR**						
Mean (SD)	0.52 (0.12)	0.49 (0.09)	0.59 (0.15)	0.55 (0.12)	0.53 (0.09)	0.62 (0.12)
Median (IQR)	0.51 (0.43–0.57)	0.49 (0.42–0.55)	0.56 (0.50–0.66)	0.53 (0.48–0.64)	0.49 (0.45–0.58)	0.59 (0.52–0.65)
**AR-HGS (kg)**						
Mean (SD)	26.91 (12.19)	30.55 (11.72)	20.50 (10.43)	21.75 (11.37)	21.24 (8.33)	12.51 (6.87)
Median (IQR)	27.25 (16.37–36.12)	31.50 (21.25–40.25)	20.50 (11.00–31.50)	18.25 (11.38–23.68)	22.35 (15.12–26.00)	12.55 (5.50–19.62)
**AL-HGS (kg)**						
Mean (SD)	26.22 (11.86)	30.14 (11.35)	19.31 (9.51)	20.60 (11.10)	19.66 (7.53)	11.15 (6.24)
Median (IQR)	25.75 (16.37–34.50)	29.50 (22.00–39.00)	18.00 (10.50–27.00)	16.95 (10.00–21.85)	19.00 (14.00–23.75)	10.75 (4.87–16.50)
**RR-HGS **						
Mean (SD)	0.40 (0.20)	0.47 (0.19)	0.29 (0.15)	0.34 (0.18)	0.36 (0.14)	0.20 (0.11)
Median (IQR)	0.35 (0.24–0.55)	0.47 (0.32–0.60)	0.25 (0.17–0.38)	0.26 (0.21–0.40)	0.34 (0.23–0.44)	0.21 (0.09–0.25)
**RL-HGS **						
Mean (SD)	0.40 (0.20)	0.46 (0.19)	0.28 (0.16)	0.32 (0.18)	0.33 (0.12)	0.17 (0.09)
Median (IQR)	0.36 (0.24–0.50)	0.46 (0.31–0.56)	0.24 (0.17–0.36)	0.26 (0.17–0.36)	0.31 (0.33–0.44)	0.17 (0.07–0.25)
**CMJ (cm)**						
Mean (SD)	14.08 (6.73)	16.71 (6.08)	10.20 (4.26)	14.09 (7.33)	16.64 (6.44)	9.59 (6.71)
Median (IQR)	13.52 (9.04–18.65)	17.20 (12.47–21.54)	10.55 (7.48–13.30)	13.32 (8.12–19.90)	15.86 (11.79–21.52)	8.20 (3.78–15.79)
**TUG (s) **						
Mean (SD)	6.12 (1.88)	6.06 (1.90)	6.21 (1.90)	6.90 (2.12)	6.99 (2.03)	8.28 (2.02)
Median (IQR)	5.45 (4.69–7.32)	5.40 (4.69–7.17)	5.51 (4.59–7.52)	7.59 (6.29–8.97)	7.09 (5.02–8.16)	8.39 (6.62–9.67)
**5STS (s) **						
Mean (SD)	9.69 (2.63)	9.27 (3.29)	10.46 (2.35)	9.61 (2.64)	9.44 (2.58)	9.91 (2.79)
Median (IQR)	9.00 (7.41–11.07)	8.48 (7.04–10.41)	10.86 (8.49–11.58)	9.13 (7.58–11.49)	8.97 (7.53–11.45)	9.59 (8.16–11.57)
**Agility 4 × 10 m (s)**						
Mean (SD)	17.82 (4.76)	16.81 (3.96)	19.60 (5.59)	18.57 (4.73)	17.74 (4.25)	21.26 (4.50)
Median (IQR)	16.68 (14.13–19.51)	16.71 (13.83–18.82)	16.76 (15.75–24.20)	18.50 (16.25–21.33)	17.31 (14.96–19.87)	19.99 (18.50–22.86)
**ARS (m/s) **						
Mean (SD)	2.38 (0.53)	2.49 (0.50)	2.18 (0.53)	2.28 (0.52)	2.37 (0.52)	1.95 (0.33)
Median (IQR)	2.39 (2.04–2.82)	2.39 (2.12–2.89)	2.40 (1.65–2.53)	2.16 (1.88–2.46)	2.31 (2.01–2.67)	2.00 (1.74–2.16)

BMI = body mass index; WC = waist circumference; WHtR = waist-to-height ratio; AR-HGS = absolute right handgrip strength; AL-HGS = absolute left handgrip strength; RR-HGS = relative right handgrip strength; RL-HGS = relative left handgrip strength; CMJ = countermovement jump; TUG = time up and go test; 5STS = five repetition sit to stand test; ARS = average running speed.

## Data Availability

The data that support the findings of this study are available from the corresponding author upon reasonable request.
